# Comparing the segmentation of quantitative phase images of neurons using convolutional neural networks trained on simulated and augmented imagery

**DOI:** 10.1117/1.NPh.10.3.035004

**Published:** 2023-06-30

**Authors:** Eddie M. Gil, Zachary A. Steelman, Anna Sedelnikova, Joel N. Bixler

**Affiliations:** a Texas A&M University, Department of Biomedical Engineering, College Station, Texas, United States; b SAIC, JBSA Fort Sam Houston, Texas, United States; c Air Force Research Laboratory, JBSA Fort Sam Houston, Texas, United States

**Keywords:** quantitative phase imaging, deep learning, neuronal growth simulations

## Abstract

**Significance:**

Quantitative phase imaging (QPI) can visualize cellular morphology and measure dry mass. Automated segmentation of QPI imagery is desirable for tracking neuron growth. Convolutional neural networks (CNNs) have provided state-of-the-art results for image segmentation. Improving the amount and robustness of training data is often crucial to improving CNN output on novel samples, but acquiring enough labeled data can be labor intensive. Data augmentation and simulation can be used to address this, but it is unclear whether low-complexity data can result in useful network generalization.

**Aim:**

We trained CNNs on abstract images of neurons and on augmented images of real neurons. We then benchmarked the resulting models against human labeling.

**Approach:**

We used a stochastic simulation of neuron growth to guide abstract QPI image and label generation. We then tested the segmentation performance of networks trained on augmented data and networks trained on simulated data against manual labeling established via consensus of three human labelers.

**Results:**

We show that training on augmented real data resulted in a model that achieved the best Dice coefficients in our group of CNNs. The largest percent difference in dry mass estimation with respect to the ground truth was driven by segmentation errors of cell debris and phase noise. The error in dry mass when considering the cell body alone was similar between the CNNs. Neurite pixels only accounted for ∼6% of the total image space, making them a difficult feature to learn. Future efforts should consider methods for improving neurite segmentation quality.

**Conclusions:**

Augmented data outperformed the simulated abstract data for this testing set. The quality of segmentation of neurites was the key difference in performance between the models. Notably, even humans performed poorly when segmenting neurites. Further work is needed to improve the segmentation quality of neurites.

## 1 Introduction

Precise tracking of cell morphology over time is highly desirable for basic science.^1^^,^^2^ This is particularly true of neurons that exhibit intricate branching structure driven by their essential function as bioelectric signal transmitters. Classical measurement of neuronal growth generally involves fluorescence labeling and identification of one or more parameters, such as dendritic length or distribution of segments. However, there is an emerging understanding that more complete representations of cell and network morphology are required to fully capture the complex geometry and connectivity of multi-cell systems.^3^^,^^4^

An attractive path toward improved measurements of neuron growth is quantitative phase imaging (QPI) combined with convolutional neural networks (CNNs) for semantic segmentation. QPI is a label-free microscopy technique that creates images by measuring the optical pathlength difference at each point in a sample.^5^ Critically, it also enables the computation of cellular dry mass (i.e., the total non-water mass of cellular components) by integrating the phase over the area encompassed by the cell or subcellular component of interest.^6^ This measurement is highly useful since it is an independent and precise measurement of cell growth and metabolism, which is not available from fluorescence-based images.^7^ Automated segmentation of QPI images using deep learning would immediately identify the mass distribution and morphology of the neuronal network and could be used to rapidly process large batches of images.

High-quality deep learning models have been trained to segment various cell types, across many imaging modalities^8^^–^^10^ including QPI.^11^ Unfortunately, deep learning models require thousands of labeled samples to gain generalizability. In our application, collection and labeling of thousands of QPI images is a demanding task, which would also necessitate the capture of variations in cell morphology, system magnification, field of view, and system noise characteristics. Moreover, simply increasing the number of training samples is not enough to design a robust network. The distribution of outputs from deep learning models tends to bias toward sample classes and feature sets that are overrepresented in the training data.^12^^,^^13^ In cases where subject data fail to capture the sources of variance expected in practice, acquiring additional training data does not always improve model performance.^14^

One way to address these issues is to pretrain a model with known benchmark data and then fine tune on a dataset that more closely represents the desired task.^15^^,^^16^ This approach is most useful when the source and target data contain similar patterns.^17^ Cellpose, a generalist model for cell segmentation, takes this approach, and incorporates cell images from brightfield microscopy and fluorescence microscopy, as well as non-microscopy images.^18^ While this model is highly effective at whole cell segmentation, and cytoplasm and nucleus segmentation, there are a few details that make it insufficient for our application. First, QPI images are not present in the training data, and thus QPI-specific noise sources and imaging aberrations are not captured by Cellpose’s dataset. Second, QPI is a projection-based imaging tool, so cytoplasm and nuclei cannot be separated to provide targets for segmentation in Cellpose. Finally, dry mass calculations for measuring growth dynamics require differentiating among the cell body, neurites, and the background segmentation masks. Thus there is a need to train a model to segment QPI images of neurons.

An alternative solution to utilization of existing generalist models is to use data augmentation to expand the pool of available training data. Generative adversarial networks can do this by learning to generate realistic training samples, but fine control over the feature content of the generated data is challenging.^19^^,^^20^ Data augmentation can provide such control and is performed with techniques, such as affine transforms, pixel gamma variation, non-linear image warping, and image mixing via cropping and summing operations.^21^ Typically, only transforms, which preserve the relationship between the training data and labels, are selected, since failure to do so can lead to worse model performance. Augmentation is thought to work by having the network learn to average across orbits of data with larger variance than would otherwise exist without augmentation. This results in model invariance to transforms in the data augmentation policy.^22^ Although incredibly effective, determining the most effective augmentation policy for a particular task requires some tuning.

Regardless, deep learning models do not generally handle abstraction well, which leads to poorer performance on novel data.^23^^,^^24^ Fortunately, abstract concepts are often based on a simple set of rules along with a high degree of variance.^25^^–^^27^ A stochastic model may provide the solution. In our case, a simulated model of neurons growing could provide images with more variance than augmenting a finite set. This approach also has the benefits of requiring zero training data or manual labeling and high tunability for various related tasks.

In this study, we tested the effectiveness of neuron segmentation models trained on various types of data. In particular, we trained our networks on abstract simulated data, as well as augmented data, and compared the results to a known generalist cell segmentation model, and to human labelers. We implemented a 2D version of NETMORPH,^28^ a known stochastic biological model for neuron growth as the generator for our abstract training data. We generated a dataset of 5000 realistic QPI images of neurons. Labels were automatically generated for these images during the simulation process, and Gaussian speckle noise was added to approximate real-world imaging. We reserved 10% of the data to check for overfitting and trained on the remaining 90%. We trained a U-Net^29^ with residual units^30^ to segment images of neurons into cell bodies and neurites. We also included results from various formulations of data augmentation to compare the network output between an entirely simulated training set, and one which has been augmented from a small number of real images. Although abstract data were not as effective as augmentation, it may be beneficial in certain scenarios. We believe that our study serves as a useful examination of semantic segmentation training methodologies.

## 2 Methods

### 2.1 Considerations for Simulating Images with Patterns Similar to Lab-Acquired QPI Images

The goal of the simulation step was to generate training data with patterns similar to those contained in the lab-acquired images, while staying abstract in nature. In lab-acquired QPI images, a cell is an object with specific shape, phase intensity, and texture-based features. The research focused primarily on simulating relevant geometric and biological features. Although this limited the number of patterns that the training dataset contained, these features allowed for effective segmentation.

Prior to updating the neuron growth simulation from our preliminary work,^31^ a small set of 10 QPI images of neuroblastoma and glioma hybrid NG108-15 cells was acquired. These cells were grown and imaged independently of the final testing set acquired to validate the segmentation results. The QPI system^6^ was modified to utilize a 10× objective lens (Olympus UPlanFLN, 10×/0.30 NA) and a sensor (FLIR Blackfly S) with 3.45-μm square pixels. Because of the 10× objective, each pixel covered 0.345  μm. Sub-frames of 1024×1024  pixels were acquired from the central position of the sensor where the resulting image had a field of view of 353.3×353.3  μm.

To match these parameters, the simulated neurons were generated in 512×512-pixel array unsigned 8-bit integers (uint8) and saved as portable network graphics (PNG) image files. Additionally, all lab-acquired images were resized to 512×512  pixels to match the input size of the training data. Resampling the PNGs in this way eased the computational burden when the CNN was in use. This was acceptable in the imaging system, as the diffraction-limited spot size of ∼1.3  μm was still effectively sampled under the Nyquist criterion after downsampling. Hand labeling was performed on all lab-acquired images. Ground truth labels were generated using the consensus of masks obtained from three human labelers. The 10 preliminary images were excluded from the testing set, since they were used to determine the approximate range of cell sizes. A region finding algorithm was used with the binarized image labels to isolate the cells and assign a rectangular bounding box to the cell body portion of the cells in the 10 preliminary images. The major and minor axes of the cell body contained in each bounding box were recorded. The 10 images were also the source images for the augmentation-generated training data.

### 2.2 Simulating Neurons

We implemented a variation of the NETMORPH model outlined by Koene et al.^28^ The model has the ability to stochastically generate simulated “Petri dishes” of neurons within a defined field of view by assuming parameters for iterative neuron growth. At each time step, the model evaluates probabilistic functions controlling for elongation, neurite branching, and directional change of neurite growth cones.

The program generated a list of points in x and y that defined placement of line segments representing discretized fractions of neurite into a 353.3×353.3-μm region. The images were uint8 arrays with pixel values between 0 and 255. The line segments were 1-pixel wide rectangles with image intensity of 255. A total of 200 Petri dishes with 25-time steps of simulated neuron growth were generated. For each dish, a maximum of 10 neurons were simulated, with a minimum of one neuron per dish. Each input image was normalized by its minimum and maximum so that it had value between 0 and 1 prior to being processed by the network.

#### 2.2.1 Simulating cell bodies

Ellipsoids were used to simulate cell bodies and were placed at the point where the neuron growth simulation begins the initial segment for each neuron. Each ellipsoid was generated using the following equation:

$$z=\{\begin{array}{ll} \sqrt{r^{2}-(ax^{2}+by^{2})}, & \text{if}\;(ax^{2}+by^{2})<r^{2}\\ 0, & \text{if}\;(ax^{2}+by^{2})\geq r^{2} \end{array},$$ (1)

where r equals 1, and a and b represent scaling factors in x and y dimensions, respectively. The generated ellipsoids were rotated by an angle between 1 deg and 360 deg. Uniformly distributed random numbers controlled the radii in x and y, and the angle of rotation of the ellipsoid. Following rotation, each ellipsoid was iteratively added into a 353.28×353.28-μm array of zeros. If it was not the first neuron cell body in the simulated space, the ellipsoid was summed with the previous image held in memory, until all neurons were initialized. This occasionally created the realistic scenario of cell crowding or overlapping, which can increase the phase signal in QPI, and further allows for non-ellipsoid shapes to be represented. The achieved diameters in x were between 0.69 and 138.69  μm, with an average diameter of 29.57±19.60  μm. The diameters in y were between 0.69 and 186.99  μm, with an average diameter of 31.05±23.05. The resulting diagonal lengths were between 0.97 and 230.36  μm, with an average length of 43.56±29.26  μm.

**Fig. 1:**
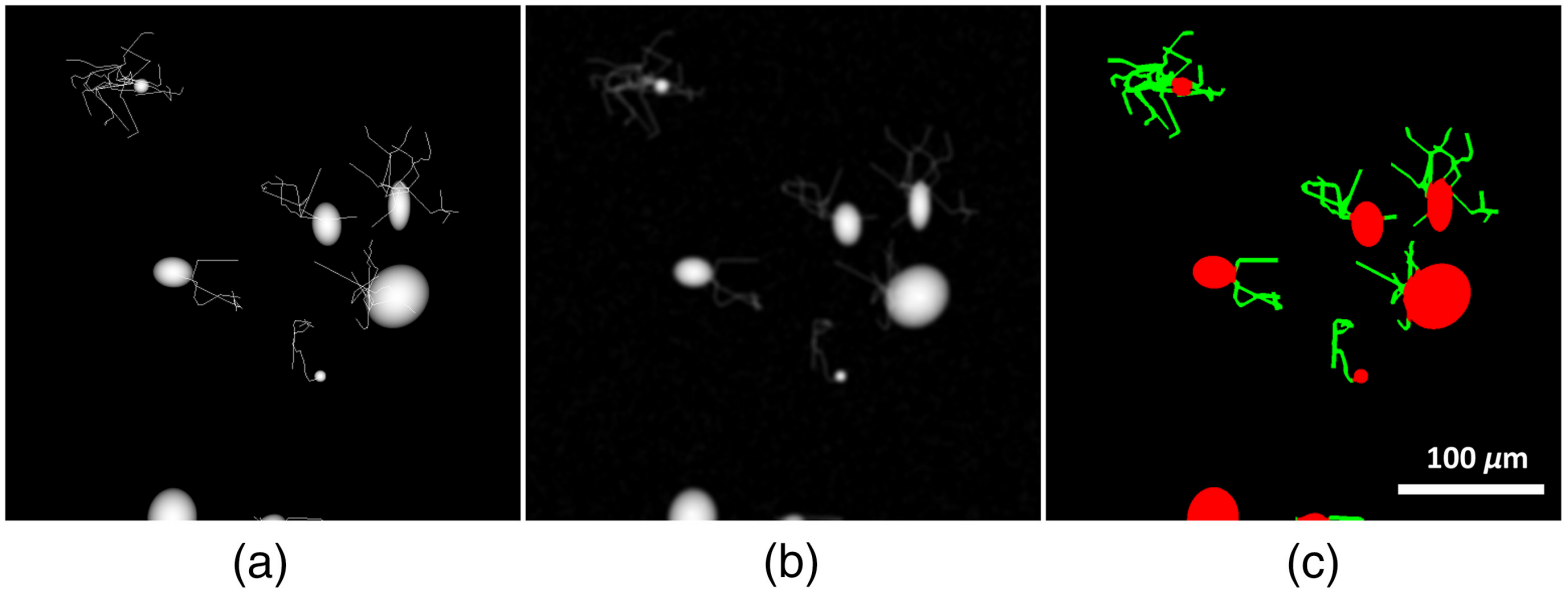
(a) Ball and stick approximation of neurons with neurite locations guided by output points from a neuronal growth implementation. (b) Result of transforming the ball and stick approximation into a pseudo-QPI image. (c) Label image where red pixels mark cell body, and green pixels mark neurite.

Each image was normalized with respect to its maximum prior to saving in order to avoid clipping at 255. The neurite line segments were only added into the image after all ellipsoids had been placed. An example of these images is depicted in Fig. 1.

#### 2.2.2 Smoothing the resulting QPI image and adding noise

The images resulting from Sec. 2.2.1 were blurred using a Gaussian filter with kernel size 21×21 and $\sigma_{x}$ and $\sigma_{y}$ of 21 pixels. This smoothed the transition between the simulated neurites and cell bodies and approximated the phase profiles of living cells. In living cells, the phase signal is thickest in the middle of the cell body and drops to zero at the edge of the cell in accordance with the physical thickness at each spatial location. Blurring also ensured that the thin neurites exhibited low-phase signal compared with the cell body, as these thin protrusions of the cell exhibit minimal phase signal.^32^

Finally, synthetic noise was added to the smoothed images to approximate the behavior of phase and speckle noise, along with cell debris commonly observed in QPI images of neurons. To add such noise, a matrix of uniformly distributed random numbers between 0 and 1 was generated. Numbers above 0.95 were set to 255, whereas numbers below 0.95 were set to 0. The matrix was then blurred by a series of Gaussian filters with size 3×3, then with size 11×11, then size 31×31. A new sample of noise was generated for each of the simulated QPI images. After the noise was added, the final output was blurred with a small Gaussian filter to blend all of the parts together.

#### 2.2.3 Automatically generating labels for the set of simulated neurons

Labels for the image were created by assigning cell body pixels the color red, neurite pixels the color green, and background pixels the color black. On the first iteration of neuron generation after the ellipsoids were placed to represent the cell body, and after the QPI transformation was applied, thresholding was done such that all the pixels with value above the noise floor are labeled as cell body. When a new line segment was added into the image, the segment was labeled as neurite. Figure 1 shows a sample of the simulated abstract data which we refer to as ball and stick images. The corresponding smoothed image and its resulting label are shown in Figs. 1(b) and 1(c), respectively.

**Fig. 2:**
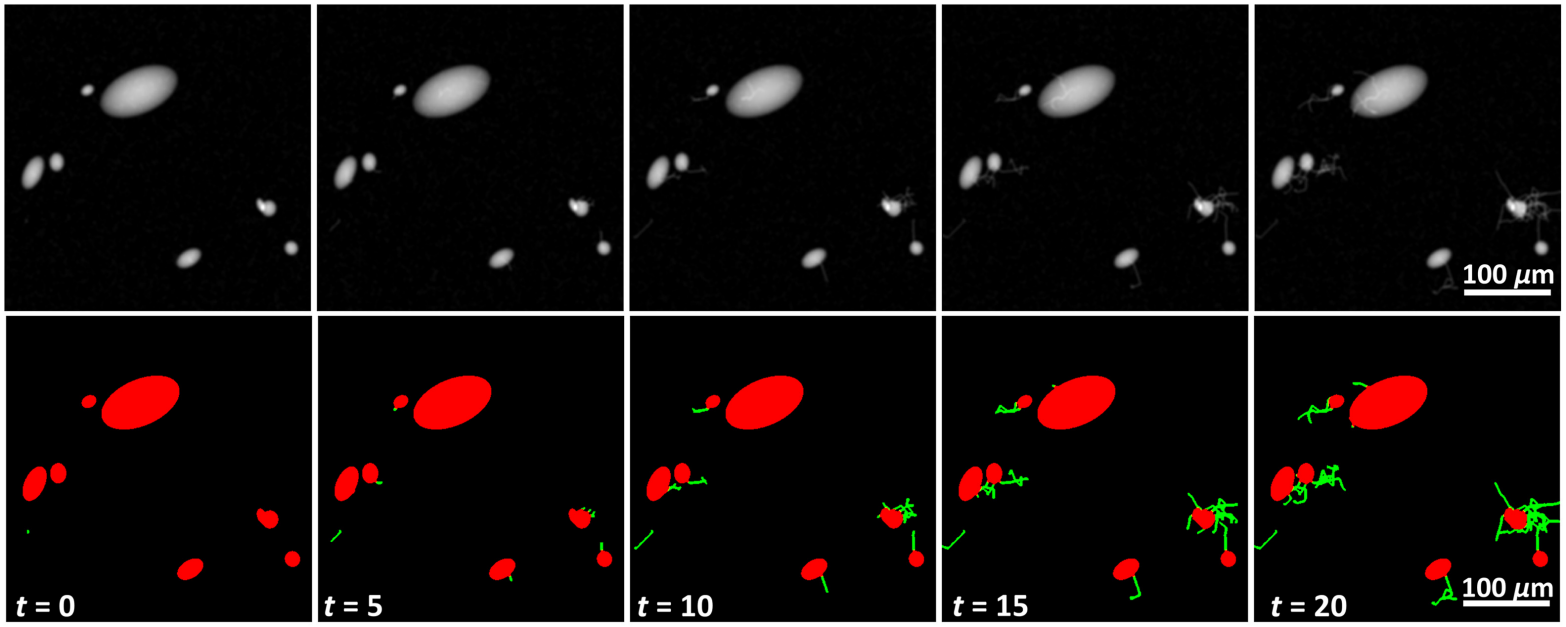
Samples of neuron growth at five time points, along with their respective masks. There were regions of increased pixel intensity at locations where cell bodies overlapped with each other, and in regions where neurites overlapped with cell bodies. This added some variability in the image intensity, which was needed since the texture information in the simulated images was limited to smooth surfaces.

Figure 2 shows simulated neurons along with their masks at various time points during the neuron growth simulation. When the cell bodies overlapped, they created spaces with slightly brighter intensity than other ellipsoid bodies. The increased brightness was consistent with some of the texture information from overlapping cell bodies present in the lab-acquired images. Also the regions of overlap helped the network become sensitive to more complex shapes which were the result of summing between ellipsoids. Pixels where the cell body covered the underlying neurite were given the label of cell body, which added some texture information to the cells. Although the neurites were thin, they covered thicker areas as they grew, which helped the network be sensitive to a wide range of neurite sizes.

### 2.3 Network Architecture and Training Details

**Fig. 3:**
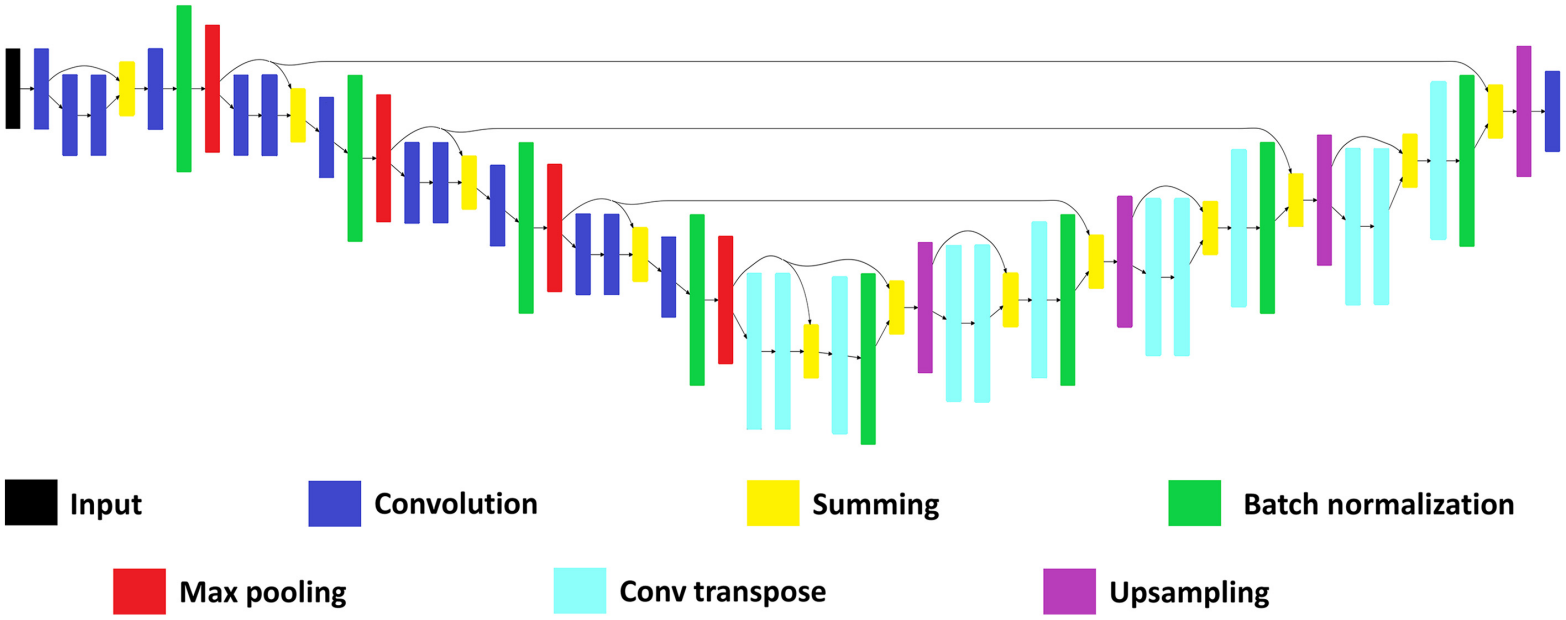
Our model is a modified version of a U-Net that uses residual blocks in the intermediate stages.

The neural network architecture used to address this segmentation problem was a modified version of a U-Net.^29^ The architecture is shown in Fig. 3. The model included residual blocks as the intermediate stages as opposed to single layers of convolution. This helped to increase the capacity of the network over the base U-Net model. Each convolutional layer made use of 16 filters of size 3×3. The summing blocks reduced the number of channels in their output to 1, while the following Conv2D or Conv2DTranspose increased the number of filters back to 16. All layers except for the final layer used rectified linear units for their activation. The final layer used SoftMax activation. All max pooling and upsampling layers were of size 2×2, so that the image at those points was either halved or doubled in size. The architecture was initialized from scratch for each trained model. Glorot uniform parameter initialization was used for each layer since this was the default parameter initializer for Tensorflow Keras layers. Each network was trained for 200 epochs on its respective training dataset. Stochastic gradient descent (SGD) was used to optimize the networks with a batch size of five images. The learning rate was set to 0.01. All other hyperparameters for the SGD optimizer were left as the default settings in the TensorFlow Keras optimizer method. The network had a total of 56,787 parameters, with 56,531 trainable and 256 nontrainable. It took 9.5 h to train the network using a laptop with a NVIDIA GeForce 1060 GPU. Since the network had to pass through 5000 images on every epoch, each image on average took 0.036 s to process.

Loss based on the Dice coefficient was used to train the network. The Dice coefficient D is given by the following equation:

$$D=\frac{2*\sum y_{\text{true}}*y_{\mathrm{pred}}}{\sum y_{\text{true}}^{2}+\sum y_{\mathrm{pred}}^{2}},$$ (2)

where $y_{\text{true}}$ and $y_{\text{pred}}$ are vectors containing all of the pixels in the ground truth image and in the network output image, respectively. Since the network output and the labeled image are both three-channel images, they are flattened into 1D vectors before being passed to a function implementing Eq. (2). The loss function is then

loss=1−D. (3)

It is important to note that the Dice coefficient, while considered a measure of overlap, does not have dependence on the location of particular pixels. Instead, it more closely describes how well the pixels in $y_{\text{pred}}$ match the values of the corresponding pixels in $y_{\text{true}}$. The Dice coefficient between the network outputs was computed from the set of 112 lab-acquired QPI images of NG108 neurons, and their manually segmented counterparts from three human labelers, in order to validate the performance of the networks on real-world data.

### 2.4 Clearing Erroneous Segments with Size Filtering

Occasionally, the network trained on simulated data would improperly segment small phase objects, such as cell debris as either neurite or cell body. To correct for this, a region size filter was added to the output of the network. First, the network output was binarized so that background pixels were assigned a value of 0 and all other pixels were assigned a value of 1. A region was then defined as a collection of pixels with a value of 1 that are adjacently connected to at least four other pixels with value of 1. Then the area of each region in an image was computed and any regions smaller than $714\;\mu m^{2}$ were removed. The area threshold was determined by size filtering the lab acquired validation images at thresholds from 119 to $1071\;\mu m^{2}$ at $119\;\mu m^{2}$ increments. The threshold that resulted in the highest overall Dice coefficient was selected. The resulting size filtration step reduced the number of false positive regions and ultimately improved the visual appearance of the final network output by eliminating a number of random regions associated with either cell debris or noise which were improperly classified as cellular components.

### 2.5 Lab Acquisition of QPI Images

A quantitative phase microscope, described in previous publications,^6^ was utilized for image acquisition. Briefly, a supercontinuum laser (Fianium, NKT Photonics) was filtered to a low coherence (λ=633  nm and Δλ∼1  nm) to provide sufficient interference while minimizing speckle noise. The filtered beam was polarized using a linear polarizer and split between sample and reference arms. In the sample path, the collimated beam was passed through the neurons, collected using a wide-field objective lens (Olympus, 10×, NA = 0.3), and focused onto the CMOS detector (FLIR, 3.45 mm pixels) using a 2 in. achromatic tube lens with focal length 180 mm. In the reference arm, the beam was expanded to encompass the spatial extent of the detector plane using a 4f telescope, and a half-wave plate was used to match polarization between the two paths, maximizing fringe visibility. The sample and reference beam were recombined at a slight angle using a 2 in. beam splitter cube, and a delay arm in the sample path was used to precisely match the two arms in optical pathlength.

Each interferogram was acquired with an integration time of 15 ms. Once acquired, the images were processed according to standard protocols.^33^ The raw image was subjected to a fast Fourier transform, and the off-axis term of interest was isolated using a spatial filter. This term was inverse-transformed, and a phase-unwrapping algorithm was applied to remove any 2π ambiguity. The background was manually segmented and detrended to provide a flat phase field.

### 2.6 Data Augmentation Procedure

A sample of 10 neuron images independent from the final testing set were collected to be used as the source data for the augmentation policy. The augmented training dataset was created by randomly selecting an image from this small set and applying transforms to the image. Size rescaling was performed by sampling a normal distribution (μ=1, σ=0.25), which was constrained to the range 0.5 to 1.5. The scaling factor was multiplied by the original dimensions of the image (512×512) to determine the region of interest. When the scaling factor was greater than one, zeros were padded onto the image to reach the desired output size, and then the resulting image was resized to 512×512. This effectively shrinks the QPI portion of the image and makes the neurons appear smaller than their original size. When the scaling factor is <1, then a region of the size scaling factor multiplied by (512×512) was randomly selected from the image and cropped. That region is then resized to 512×512, which results in neurons that appear larger than they are. At scaling factor of exactly one, the image was left unchanged. While U-Net is able to learn features at various scales due to its downsampling and upsampling layers, in our implementation, these layers are strictly implemented in factors of 2. Scaling the neurons in a normal distribution allows for better coverage of the natural variability in cell body and neurite sizes.

Following rescaling, the images were randomly flipped. The flipping options were left–right, up–down, left–right + up–down, and no flip. Each option occurred with equal probability. After flipping, the images were randomly rotated 0, 90, 180, or 270 deg with equal probability. The same size scaling, flipping, and rotations were applied to the labels, so that the training sample and the training label maintained their 1-to-1 relationship. Only the training images and not their labels were gamma scaled by first normalizing the image from 0 to 1, then raising the image to the power of g, where g is a normally distributed number (μ=1, σ=0.35) constrained to be between 0.7 and 1.7. Although this gamma scaling is asymmetric, it prevents the training image from oversaturating or undersaturating.

The augmentation policy was applied to the sample of 10 images and run until 50,000 data points were generated. It is important to note that while data augmentation can be implemented in the data loading step to allow for new augmented data on each batch, we augmented prior to any training to keep a consistent training dataset. This ensured that any differences in performance were not due to differences in the realized augmentation images. A random subsample of 5000 images was used as training data. A second subsample of 25,000 images was used to determine whether benefits were due to only increasing the number of training samples, and if there was a limit to how much improvement can be achieved when augmenting from only 10 images. Next, to determine whether a mixed random sample of real and simulated images could be beneficial after augmentation, two such additional training datasets were created. The first set was made by applying augmentation to a combination of the 10 real images with 10 ellipsoid images, whereas the second set was made by augmenting another combination of the 10 real images with 90 ellipsoid images. These sets were made so that the resulting set was 50,000 images, and subsamples of 5000 images were randomly selected for training.

### 2.7 Cell Culture

Neuroblastoma and glioma hybrid NG108-15 cells were cultured according to the manufacturer’s specification at 37°C with 5% ${\mathrm{CO}}_{2}$ in air with a relative humidity of 95%. Cells were harvested between passages 20 and 30 and plated at a density of $10^{4}\;\text{cells}$ per square centimeter. Cells were plated in 35 mm glass-bottomed dishes coated with poly-d-lysine. Differentiation was induced 24 h after plating by replacing the growth medium with a differentiating medium, in which the fetal bovine serum had been replaced with B27 (Gibco 17504044) and supplemented with 1 mM dibutyryl cyclic AMP (Tocris 1141). The differentiating medium was replaced every 48 h, and cells were imaged after 7 to 14 days of differentiation. The cells were removed from the incubator and imaged in their culture medium. Cells were removed from the incubator for no longer than 5 min during image acquisition.

To match the data range of the simulated images, all pixels with negative phase values were set to 0. This was acceptable, as these were generally background pixels within the noise floor, which had been background-subtracted such that the average background phase signal was 0 rad. The minimum pixel value was then subtracted from the image. The result was then divided by its maximum value. The final image was then multiplied by 255 and saved as a uint8 image. When processed by the network, these images were divided by 255 so that they had value between 0 and 1.

### 2.8 Human Labeling of QPI Images and Generation of Ground Truth Labels

The ground truth segmentation labels for the validation set were created using an agreement comparison scheme generated using labels from three human labelers. Each human labeler used a manual image editing software (Microsoft Paint 3D) to manually label the cell bodies and neurites. Within Paint 3D, human labelers were instructed to utilize the fill tool to color the cell bodies red and the neurites green. Labeling in this way allowed the labelers to save a copy of the image with cell body and neurite labels. After these labels were made, the cell debris and all other pixels were cleared by setting all pixels that were not red or green to 0. In this way, the background pixels were indirectly labeled. Red pixels were set to 255 in the red channel, and green pixels were set to 255 in the green channel since the RGB values using the native color options were not maximal.

Once each labeler finished their preliminary label assignment, the following algorithm was used to generate a consensus on the final ground truth labels. At each pixel in a given image, the label assigned by each labeler was treated as a vote. The label with a majority of votes was then assigned to that pixel. This function alone accounted for 99.79% of the pixels across all 112 images in the dataset. The remaining 0.21% of pixels, where the three labelers each assigned a different identity (cell body, neurite, and background) generally occurred very close to the edge of a thin cell body or neurite, in which the phase noise made identification difficult even for humans. Thus to ensure all pixels were labeled, each ambiguous pixel was placed at the center of a window with size 51×51  pixels. The votes from this window were pooled spatially and across labelers. Then the label with the new majority vote was assigned. This spatial tie breaking algorithm was repeated until all ambiguous pixels are filled. Occasionally, a small number of pixels remained due to a three-way voting tie, which resulted in an endless loop. Endless loops were broken for any pixels where a label agreement could not be determined after 10 iterations. Here the pixel was assigned the label of background. Section 3.2 provides further discussion on the percentages of the total pixels that were labeled based on unanimous, partial, or no agreement between the labelers, and representative images highlighting where disagreement most frequently occurred.

Once the networks were trained and the ground truth masks were established, the saved model from each epoch was used to segment the lab-acquired images in the validation set. The final layer of the network was a SoftMax layer, which means that the output has value between 0 and 1 such that the channels encoding for background, cell body, and neurite sum to 1. Since the values in each channel are not guaranteed to be 0, this can create a significant number of misclassifications on background pixels. To remove those, the output of the network was thresholded at 0.5 in each channel. An alternative method would be to pick the channel with the highest value at each pixel, but this would eliminate intermediate values in the network output that can be used to break ties in performance during validation. The thresholded output and the ground truth masks were normalized between 0 and 1 to allow compatibility with the Dice computation. The Dice coefficients between the ground truth and the network outputs were recorded.

### 2.9 Collection of Labels Using GUI Cellpose

The GUI version of Cellpose was downloaded from GitHub. All the default settings were used, except for the cell size box, and the contrast slider. The cell size box was set to 30 pixels for the first collection of masks from Cellpose, and on the second selection 100 pixels was used for the cell size estimate. The intensity slider was set so that the left arrow was all the way to the left. This should set the lower pixel intensity bound to 0. The right arrow was adjusted so that the image in the GUI matched the image in the Windows Photo Viewer. Letting the autocalibration run for the contrast adjustment would saturate the image, so it was set manually. All images in the testing dataset were scanned in this way and the masks were saved to numpy binary files (.npy file). Since the masks label each region as an integer, the Cellpose segmentation masks were binarized by setting all pixels above 0 equal to 1. The Dice coefficient with respect the ground truth data was then computed.

### 2.10 Dry Mass Computation

Although the Dice coefficient provided an excellent means of validating segmentation accuracy, our ultimate goal was to use this network to track the growth of neuronal networks over time. QPI is a well-matched imaging modality for this task, as the dry mass (the total mass of intracellular constituents, minus water) is computable from a QPI image, allowing for tracking of the growth of cell networks, and even for separately tracking the growth of cell bodies and neurites.^2^ The dry mass is given by integrating over the phase at each point in the cell using

$$m_{\text{dry}}=\iint \frac{\lambda }{2\pi \alpha }M(x,y)\varphi (x,y)dx\;dy,$$ (4)

where M(x,y) is the binarized mask of the region of interest, φ(x,y) is the phase measured at a given location, λ=632.8  nm is the wavelength of the light used to create the image, and α is a constant 0.2  ml/g.^34^ The binarized mask is the output of the network following size filtering, up sampled to 1024×1024. Each pixel at this sampling is 0.345  μm across, so dx=dy=0.345  μm. The dry mass for the whole neuron regions as well as for the neurites and cell bodies were computed individually. These were compared against the images labeled by consensus agreement from three human labelers as described above in order to assess the accuracy of the dry mass values created by the segmentation network.

## 3 Results

### 3.1 Image Labeling Time

The total time to simulate and automatically label 5000 images using the biological neuron model was ∼6  h and 20 min. Human label time varied greatly based on the image complexity. When there were few neurons in the field of view with easily distinguishable neurites, labeling took as little as 1 min. For images with many neurons of different sizes, thin neurites, and cell debris, labeling took up to 1 h. By recording the difference in time stamps for the labeled images, we found that human labelers on average required 20 min to label an image. For 112 images, this would take 37 h per labeler. Since we had 3 labelers, 111 man-h were required.

### 3.2 Human Labeler Variability

**Fig. 4:**
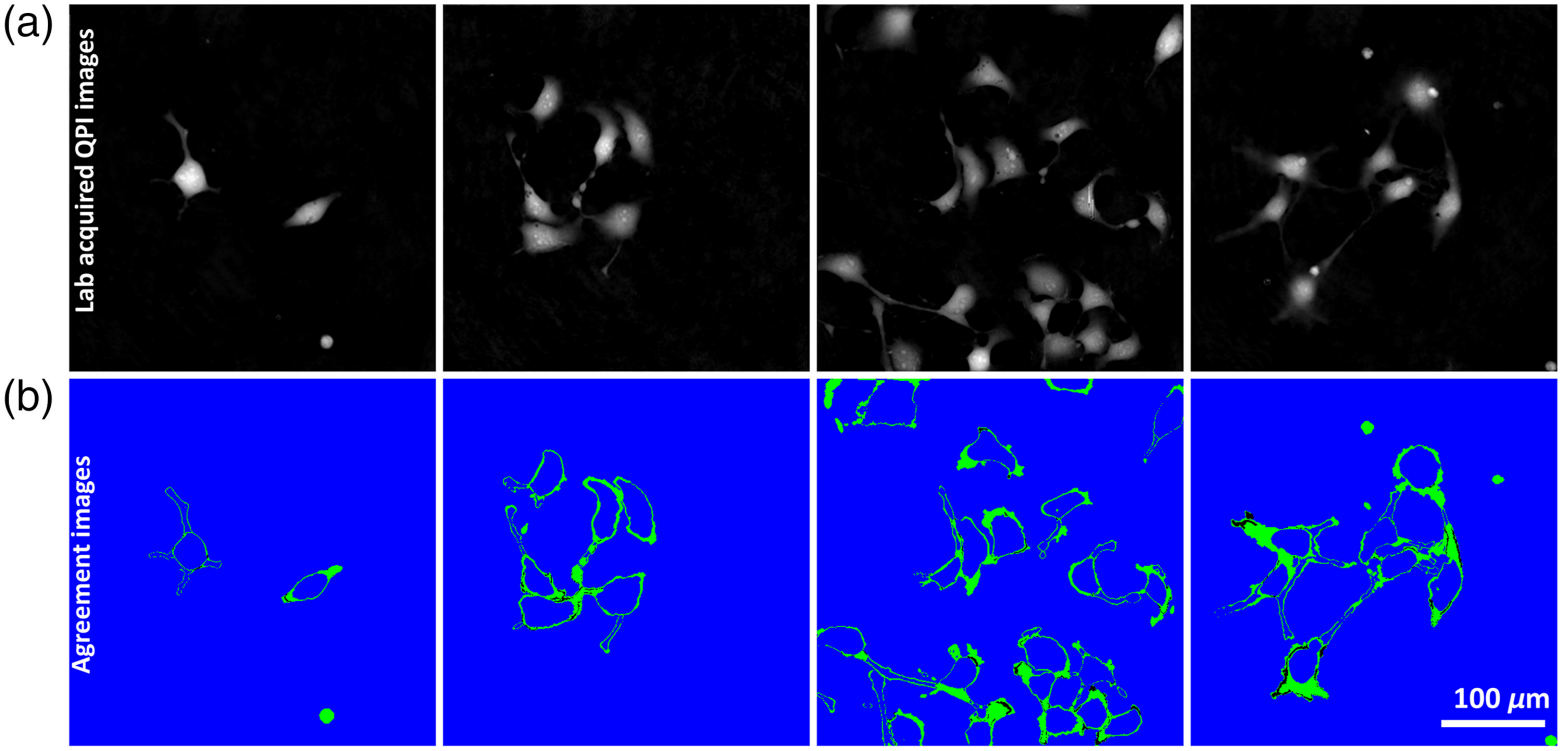
(a) Samples of lab-acquired QPI images and (b) agreement maps for the three labelers. Pixels where all three labelers agree are colored blue. Pixels where at least two of the three human labelers agree are shown in green. Pixels where there was no agreement in label are shown in black. The margins of the cell bodies and the neurites are where the highest amount of disagreement occurs.

Figure 4 shows the certainty maps for the three human labelers. The pixels where all three labelers agreed are shown in blue, whereas the pixels where two or more labelers agreed are shown in green. The pixels where there was no agreement are shown in black. For 97.35% of the total pixels in the validation set, all three labelers agreed on the label. For 2.44% of the pixels, two out of three labelers agreed, and for 0.21% no labelers agreed.

Among the three human labelers A, B, and C, labeler pair A–B achieved Dice scores of 0.80±0.10, 0.86±0.10, and 0.55±0.16, for the whole image, cell body only, and neurite only images, respectively. Labeler pair A–C achieved Dice scores of 0.84±0.06, 0.89±0.06, and 0.62±0.14, for the whole image, cell body only, and neurite only images, respectively. Labeler pair C–B achieved Dice scores of 0.84±0.0.9, 0.89±0.09, and 0.61±0.14, for the whole image, cell body only, and neurite only images, respectively.

### 3.3 Dice Coefficient Description of Network Performance with Respect to the Ground Truth Masks

Table 1 summarizes the Dice coefficients between the ground truth masks and each human labeler mask, and the Dice coefficients between the ground truth masks and the network outputs. The entries for three-channel segmentation, cell body only, and neurite only in the Cellpose categories are not given, since the cytoplasm detection model in Cellpose is only meant to provide whole-cell segmentation.

**Table 1:** Summary of Dice coefficients for all network experiments and human labeling. It is important to note that while Cellpose trained on 608 images, it makes use of 70,000 training objects. We count by images since the Dice loss function, we use aggregates loss over the entire image. Thus the independent samples are the images and not the objects.

Dice coefficient with respect to ground truth	Training set size	Cell versus not cell	Three channels	Cell body	Neurite
Human labeler-A	N/A	0.94 ± 0.04	0.89 ± 0.06	0.92 ± 0.06	0.75 ± 0.14
Human labeler-B	N/A	0.94 ± 0.09	0.91 ± 0.09	0.93 ± 0.09	0.76 ± 0.14
Human labeler-C	N/A	0.96 ± 0.01	0.93 ± 0.02	0.96 ± 0.02	0.85 ± 0.07
Augment 10 real images	25k images	0.92 ± 0.04	0.85 ± 0.06	0.89 ± 0.06	0.63 ± 0.11
Augment 10 real images	5k images	0.91 ± 0.05	0.85 ± 0.06	0.89 ± 0.05	0.58 ± 0.11
Ball and stick	5k images	0.84 ± 0.09	0.77 ± 0.08	0.87 ± 0.07	0.44 ± 0.15
Ball and stick with size filtration	5k images	0.86 ± 0.08	0.77 ± 0.07	0.86 ± 0.07	0.46 ± 0.16
Augment 0 real-10 ellipsoids	5k images	0.88 ± 0.05	0.76 ± 0.07	0.84 ± 0.07	0.44 ± 0.15
Augment 10 real-10 ellipsoid	5k images	0.91 ± 0.05	0.84 ± 0.06	0.89 ± 0.06	0.60 ± 0.12
Augment 10 real-90 ellipsoid	5k images	0.90 ± 0.05	0.84 ± 0.06	0.90 ± 0.06	0.57 ± 0.13
Cellpose-size 30	608 images	0.77 ± 0.26	N/A	N/A	N/A
Cellpose-size 100	608 images	0.74 ± 0.26	N/A	N/A	N/A

### 3.4 Network Segmentation Mask Outputs

**Fig. 5:**
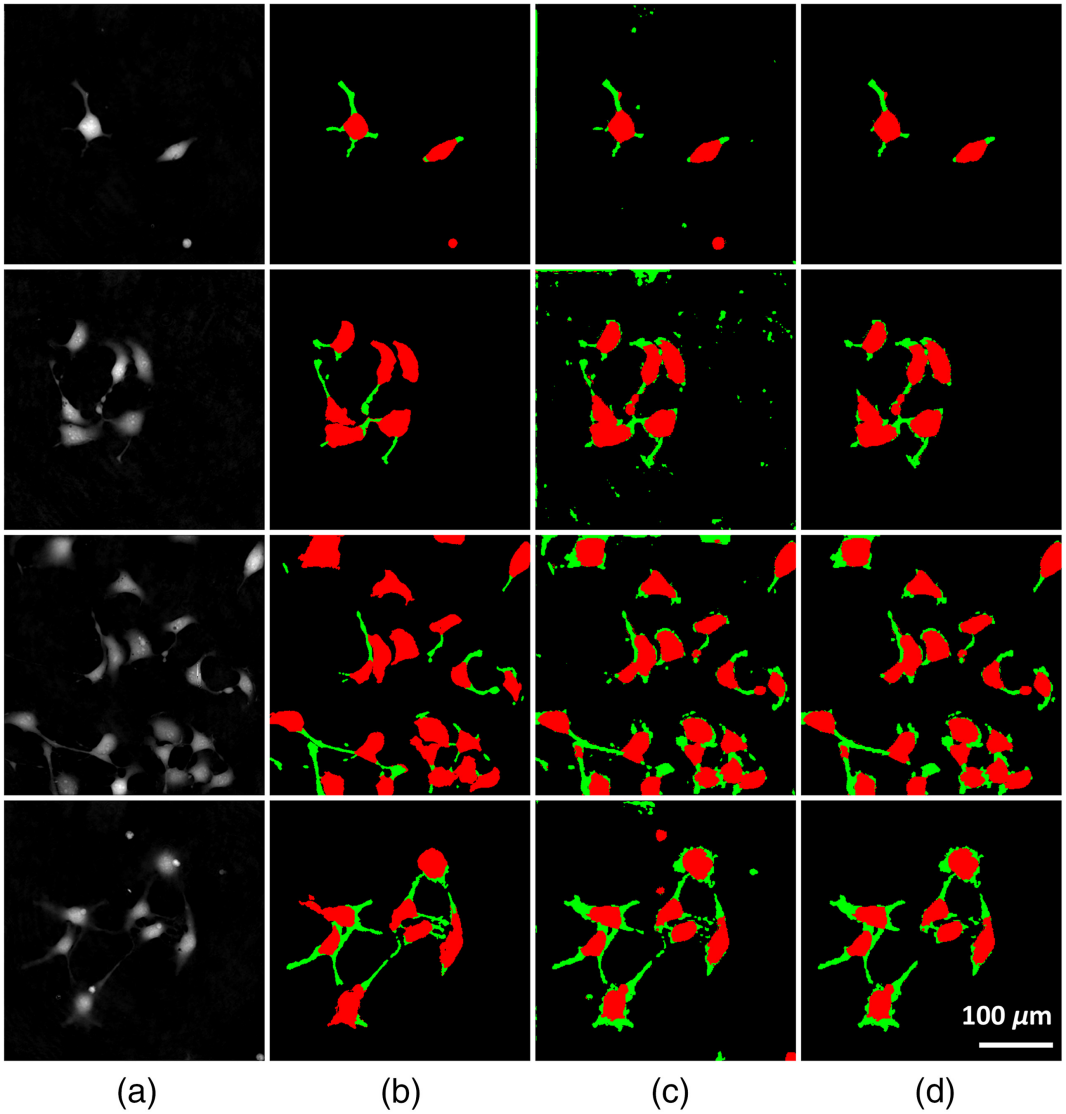
Samples of images from the validation data alongside their ground truth and the network output for the model trained on abstract data. The network is robust to the number of cells, even when this exceeds the number of potentially generated cells per Petri dish. The network is particularly well suited to detecting cell bodies. The false positives for the network are mostly due to the similar appearance between neurites and the cell debris. (a) Input images, (b) ground truth, (c) network output, and (d) size filter output.

The input, corresponding ground truth, and network output (trained on simulated data) for various QPI images, along with the mask appearance after size filtering are shown in Fig. 5.

**Fig. 6:**
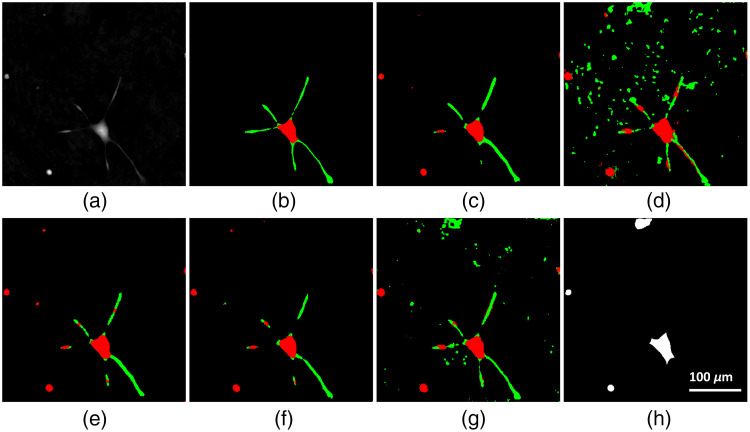
(a) A difficult QPI test image for all models; (b) the ground truth segmentation; (c) network output given a training dataset of augmented real images; (d) network output given augmented ellipsoid data: (e) network output when augmenting from a mix of 10 real and 10 ellipsoid data points; (f) network output when augmenting from a mix of 10 real and 90 ellipsoid data points; (g) network output given a training dataset of all ellipsoid data; and (h) output from Cellpose cytoplasm model.

Figures 6(c)–6(h) show the network outputs for a particularly difficult input image [Fig. 6(a)]. Figure 6(a) shows the raw QPI image, whereas Fig. 6(b) shows the ground truth masks generated from the consensus of human labelers. Although training on augmented data [Fig. 6(c)] gave the fewest false positive and false negative pixels, the abstract ellipsoid model was able to track more faint neurites [Fig. 6(g)]. But this had the drawback of increasing sensitivity to noise. Applying augmentation to the simulated ellipsoid images only [Fig. 6(d)] resulted in much more sensitivity to noise. The noise is small enough in cases like [Figs. 6(d) and 6(g)] that region size filtration can remove those pixels. When combining the real and simulated data there is increased sensitivity to neurites, but with far less false positives in neurite segmentation due to noise. Finally, our networks tend to select round areas of the cell as cell bodies, while the human labelers and Cellpose do not.

### 3.5 Dry Mass Estimates Using Network Output and Ground Truth Masks

The computed dry mass per cell, which depended on cell size, was typically on the order of a few dozen picograms to a few hundred picograms. Table 2 summarizes the percent difference in dry mass with respect to the ground truth masks for the ball and stick model, the ball and stick model following size filtration, the augmentation model, and the three human labelers. The errors reported in this table are the standard error of the mean (N=112). In each scenario, the neurites had the largest percent difference in dry mass computation, whereas the estimated cell body dry mass was closer to the true amount. This is reflective of the Dice coefficient result. Notably, the ground truth masks seem to bias toward Human C’s labels since the percent difference in dry mass error was near 0 at 0.74±0.11.

**Table 2:** Standard error of the mean on the full 112-image testing set for the ball and stick model, the ball and stick model following region size filtration, and the network trained on augmented real images, along with individual human labelers.

Mean % difference in dry mass with respect to the ground truth ± SE (N=112)	Training set size	Whole	Cell body	Neurites
Human A	N/A	2.60 ± 0.94	3.75 ± 0.96	28.73 ± 7.99
Human B	N/A	4.11 ± 1.08	6.16 ± 1.35	47.91 ± 5.26
Human C	N/A	0.74 ± 0.11	1.43 ± 0.15	15.59 ± 2.27
Augment-10 real images	25k	5.28 ± 1.08	6.78 ± 1.32	40.00 ± 4.70
Augment-10 real images	5k	5.18 ± 1.07	7.38 ± 1.45	33.38 ± 3.49
Ball and stick	5k	10.43 ± 1.34	7.60 ± 1.42	143.97 ± 15.03
Ball and stick with filter	5k	6.43 ± 1.05	7.03 ± 1.20	71.44 ± 6.68
Augment-0 real-10 ellipsoids	5k	23.81 ± 2.43	11.47 ± 1.48	463.22 ± 41.30
Augment-10 real-10 ellipsoids	5k	5.57 ± 1.11	8.54 ± 1.51	34.51 ± 3.75
Augment-10 real-90 ellipsoids	5k	5.36 ± 1.06	7.78 ± 1.45	34.29 ± 3.75

**Fig. 7:**
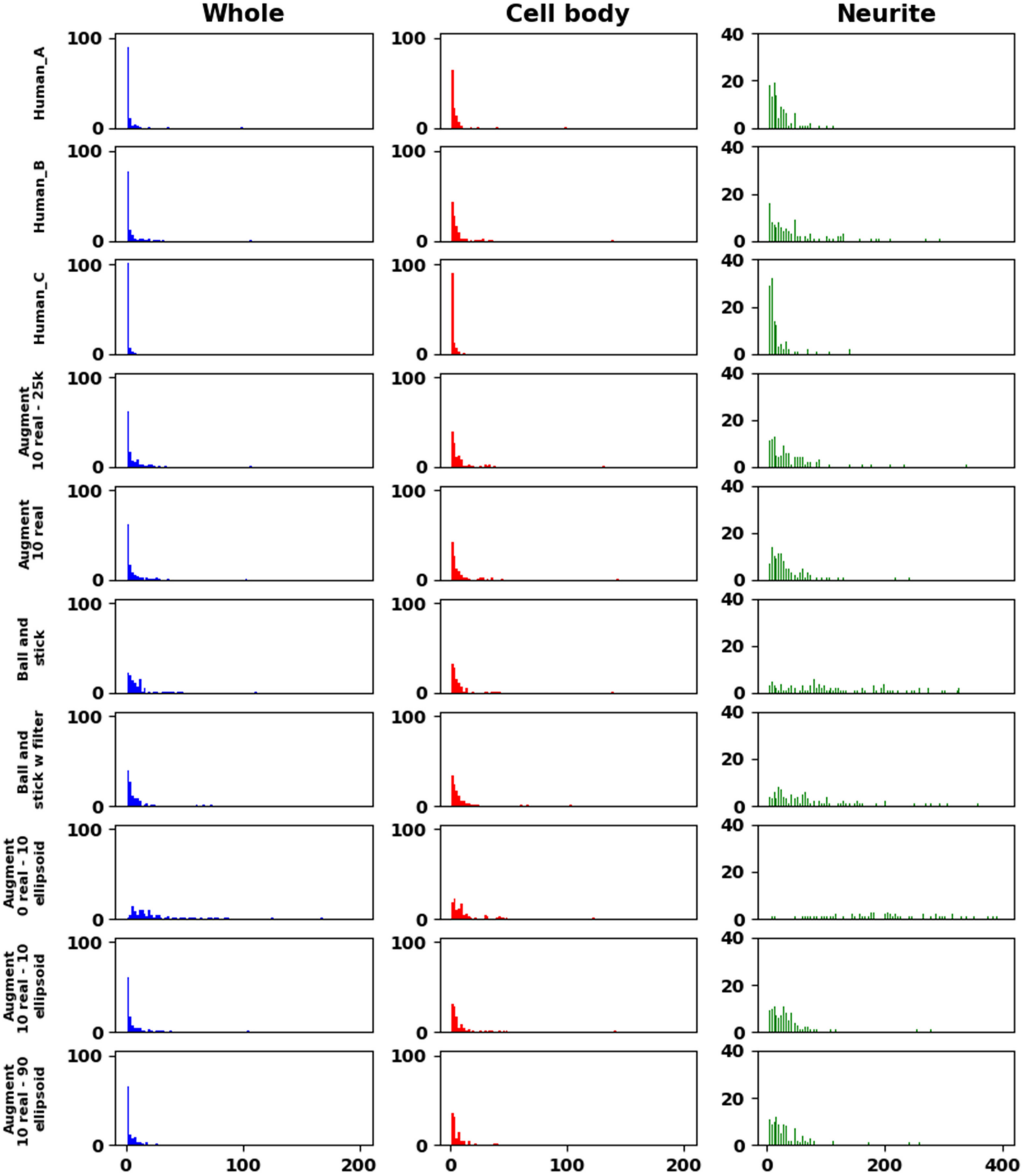
Histogram of percent difference in dry mass computed using network mask output with respect to the ground truth labels. Distribution of errors in dry mass when considering the whole cell region is similar between all three models. Most of the difference in dry mass computation occurs in the neurite regions of the cell.

Figure 7 shows histograms of the data in Table 2. The errors in dry mass estimation for the whole cell, and for the cell bodies, were typically from a few outlier images. The whole cell and cell body dry mass columns have 100 bins between 0% and 200% difference, whereas the neurite column has bins between 0% and 400% difference. The neurite dry mass in each case accounts for the largest source of error, with the ball and stick model having the widest distribution of percent difference in dry mass. However, neurite pixels only account for 5.97% of the overall pixels in the ground truth masks.

## 4 Discussion

### 4.1 Human Labeler Variability

The most likely areas of disagreement between the labelers were the boundaries between the cell bodies and the background, and the boundaries between the neurites and the background. There was some disagreement about where the cell bodies ended and where the neurites began. As each labeler used unique hardware to perform their task, differences between computer monitor color and contrast combined with the difference in labeling habits likely contributed to the disagreement in the margins of the images.

### 4.2 Dice Coefficients

Measuring the Dice coefficient provides a general sense of the degree of overlap between the ground truth labels and the model output. The percent difference in dry mass discussed in Sec. 4.4 reveals how much the degree of mask overlap resulted in mass estimation errors. Qualitative examination of resulting network masks in Sec. 4.3 reveals which inputs caused the network to fail and suggests ways to reduce the dry mass estimation errors.

Cellpose achieved lower Dice coefficients than individual human labeling, data augmentation, and the abstract model. This may be due to Cellpose not containing training data with features that were specific to QPI images, such as variance in contrast and phase artifacts from QPI imaging. As such, Cellpose had issues with false positives on areas of phase noise (unique to QPI) and with particulate matter and other cell debris. It also exhibited issues with false negatives on cells that were elongated, and on neurites, as seen in Fig. 6(h).

Augmentation of even a small amount of real QPI data provided the best Dice coefficient. Given our testing set, the model trained on the set of 5000 images augmented from 10 real images outperformed the model trained on the set of 5000 simulated images. Increasing the augmented dataset to 25,000 images did not result in performance increases compared to training on only 5000 augmented images. This makes sense given the finding that network performance scales logarithmically with data volume.^35^ Training on simulated data alone led to higher standard deviation across all segmentation groups, indicating there were likely more failure modes for these networks. This may be because the abstract model had less texture or amorphous shape information than the real data did. This can be seen in Fig. 6, where the models trained on simulated data are less robust to noise than the model trained on augmented real images. Thus improving the simulated data such that it better represents the testing cases may further improve the segmentation quality.

Neurite Dice coefficients were lower than the cell body and whole cell segmentation coefficients for each model. Even human labelers struggled to effectively delineate boundaries between neurite and background, and neurite and cell body. This is expected due to the low signal-to-noise ratios for these pixels and the lack of discrete boundaries between cell bodies and neurites. The higher standard deviation in human neurite labels implies that these labels have the highest probability of containing misspecifications due to label noise, which has been shown to result in higher testing errors.^36^

### 4.3 Discussion of Network Segmentation Mask Output

The networks were able to perform whole cell segmentation of the neurons and showed robustness to the number of cells in each image. The networks were robust to the size, shape, and number of cell bodies in the images. The models trained on ellipsoid cell bodies tended to make rounded boundaries between the cell body and neurites, rather than amorphous boundaries that the other models output. Human labeling of the cell body boundaries also varied as shown in Fig. 4. The inherent differences in labeling approaches helped to explain some of the lower cell body segmentation Dice scores.

The major difference between the segmentation models was in their sensitivity to background noise, and in the differences in how they handled the boundaries between the cell body and neurite. Unfortunately, it is likely that the gamma scaling portion of the augmentation policy caused some of the simulated noise to appear similar to the simulated neurites. Figure 6(d) reflects this since most of the false positive neurite detections were on areas of phase noise and cell debris. False positives are particularly detrimental to dry mass estimation since including extra phase signal in the dry mass integral yields higher dry mass than expected. Figure 7 shows that this is a widespread problem in the neurite segmentation task, especially for the models trained on simulated data. This points to the need for selecting and tuning the individual pieces of the augmentation policy to account for specific quirks of the data it augments. Better simulation of system noise, system background, and better neurite labels are also desirable.

### 4.4 Dry Mass

Calculating the percent difference in dry mass computed using the segmentation output of each network and the ground truth mask provides an additional metric on network performance. When considering the variability in the calculated dry mass between the three human labelers as seen in Table 2, network performance bounded by the error percentages listed here should be considered acceptable, as this would match human performance. As per Table 2, human B represents the baseline of human dry mass estimation. This labeler achieved percent differences in dry mass with respect to the ground truth of 4.11%±1.08%, 6.16%±1.35%, and 47.91%±5.26%, when considering whole cell, cell body, and neurite dry mass, respectively. As with the Dice coefficients, the neurites posed the largest challenge to effective dry mass approximation.

The model trained on 5000 augmented real images came closest to the human baseline with percent differences in dry mass with respect to the ground truth of 5.18%±1.07%, 7.38%±1.45%, and 33.38%±3.49%, when considering whole cell, cell body, and neurite dry mass, respectively. This network achieved percent differences in dry mass within 1 standard deviation of human B for both the whole cell and cell body segmentation masks. It exhibited lower neurite dry mass error than human B, but not humans A and C. The model trained on 25,000 augmented real images achieved improved cell body performance but slightly worse neurite performance. The ellipsoid model achieved percent differences in dry mass of 7.60%±1.42% and 143.97%±15.03% for the cell body and neurite masks. When using the region size filter, the ellipsoid model achieved 7.03%±1.20% and 71.44%±6.68% for the cell body and neurites, respectively. This means that training on augmented real images resulted in a segmentation model that is already within the bounds of human performance, but that the ellipsoid model requires improvement.

These networks should be generally valid for applications in monitoring cell body dry mass, and in applications where the cell body dry mass is much larger than the neurite mass, which is common given the small size and low-phase signal of neurites. However, these models would be difficult to use in applications where high sensitivity to changes in neurite dry mass is required.

### 4.5 Study Limitations

This study has several limitations. First, the testing dataset came from a single QPI microscope and a single type of neuron. Including images from more QPI systems, more variety in culture age, and various cell types may provide more insights into model failure modes.^37^^,^^38^ Recall, it has been shown that the improvement in model performance is logarithmic with data volume but large models with high representational capacity are needed to achieve this.^35^ Testing larger models with more complex architectures may result in more improvement. Moreover, the labels must have a low probability of misrepresentation of the data inputs.^36^ Thus while increasing the training and testing data, special care must be taken to reduce the probability of neurite misrepresentation.

Next, while we used a biological model of cell growth, we did not create a true physical model of the system noise or cell debris. Doing so may have made the network trained on simulated data more robust to real background noise, precluding the need for the region size filtration in that particular model. Additional strategies for segmenting subtle features should be explored in order to more effectively segment the neurites.

### 4.6 Future Work

Training on human-labeled images should be the upper bound of performance for this segmentation task. So training on real samples with data augmentation should remain the preferred approach. Refining the augmentation policies so that they are tailored to each technique and minimize distortions between the inputs and the labels may greatly improve the results as well. Additionally, techniques, such as unsupervised pretraining, allow networks to gain improved performance on a particular task by first learning to reconstruct a large amount of data that is close to the target task.^39^ In this case, it would be advantageous to understand whether the simulated neuron data could be used as a part of an unsupervised pretraining dataset, along with augmented data samples and real images. Finally, since the overall goal of this kind of segmentation work is to provide a starting point for extracting features from images, it would be beneficial to collect data showing how these different segmentation techniques impact other feature analysis like those associated with Scholl analysis.^40^

## 5 Conclusion

We tested the efficacy of abstract simulated data, and data augmentation, in training deep neural networks to segment quantitative phase images of neurons. For overall cell segmentation, abstract simulated training sets did not perform better than training on data augmented from a small sample of neuron images. Although there is comparable performance in segmentation of cell bodies between the simulated data and the augmented data, there is a clear difference in performance with regards to segmentation of neurites. We hope this study serves as a useful exploration of training methods to better understand segmentation requirements for neural imaging.

## Code, Data, and Materials Availability

The code and data used in this manuscript are not publicly available but can be obtained from the authors upon reasonable request.
